# Clinical and Immunological Profiles of HIV/AIDS Patients With First-Line Antiretroviral Treatment Failure Attending a Tertiary Care Hospital

**DOI:** 10.7759/cureus.46305

**Published:** 2023-10-01

**Authors:** Anindita Phukan, Chiranjita Phukan, Swaroop K Baruah, Diganta Buragohain, Putul Mahanta

**Affiliations:** 1 General Medicine, Institute of Digestive and Liver Disease, Guwahati, IND; 2 General Medicine, Gauhati Medical College, Guwahati, IND; 3 General Medicine, Gauhati Medical College and Hospital, Guwahati, IND; 4 Cardiology, North Eastern Indira Gandhi Regional Institute of Health and Medical Sciences, Shillong, IND; 5 Forensic Medicine and Toxicology, Nalbari Medical College and Hospital, Nalbari, IND

**Keywords:** adherence, virological failure, immunological failure, treatment failure, antiretroviral treatment

## Abstract

Objectives

Highly active antiretroviral therapy (HAART) has decreased morbidity and mortality among HIV/AIDS-infected patients; however, many patients experience treatment failure. The present study aims to evaluate HIV-infected patients' clinical and immunological profiles with first-line antiretroviral treatment (ART) failure (immunological and clinical) at tertiary care hospitals in Northeast India and explore related treatment failure factors.

Methods

The hospital-based observational study was conducted among HIV-infected patients with first-line ART failure attending a tertiary care hospital from July 1, 2019, to June 30, 2020. The type of first-line ART failure was defined as a clinical, immunological, or virological failure as decided by the State AIDS Clinical Expert Panel (SACEP) meeting. Data were analyzed with Windows MS Excel (Microsoft Corporation, Redmond, Washington) and Statistical Package for the Social Sciences (SPSS) version 21 (IBM Corp., Armonk, NY).

Results

Among the 90 HIV-infected patients experiencing first-line ART treatment failure, the majority, 38 (42.2%), were in the age group of 30-40 years, 64 (71.1%) were males, and 70 (77.8%) were of average weight. Tuberculosis was the most typical opportunistic infection, affecting 11 (12.2%) patients. Most patients (38.9%) were initially presented at clinical stage 3. Maximum failures were experienced by patients with baseline CD4 ranging from 100-200 cells/mm^3^, with 38 (42.2%) patients, and by patients on efavirenz (64.5%) and tenofovir-based regimens (56.6%). Failures occurred more for 24-30 months and were common among patients with adherence below 90%.

Conclusion

Treatment failure was more common among young male patients and those with normal body mass index (BMI). Low baseline CD4 count and poor adherence were influential in the occurrence of treatment failure. First-line ART failure was higher in tenofovir- and efavirenz-based regimens.

## Introduction

With cases documented from almost every nation, HIV infection is a global pandemic. The HIV epidemic has spread worldwide, with each wave having distinct characteristics. HIV is primarily spread through sexual contact, blood and blood products, and by infected mothers to their infants.

HIV infection can have various clinical effects. The clinical effects may be from a primary infection-related acute clinical illness through an extended asymptomatic period to advanced disease.

With an HIV/AIDS prevalence rate of 0.3%, India ranks 80th globally. It has 2.1 million individuals living with the disease, according to the 2018 Joint United Nations Program on HIV/AIDS (UNAIDS) report [1]. Antiretroviral therapy (ART) has been made available at no cost at all ART centers as part of India's National ART Program, established on April 1, 2004, under the National AIDS Control Organization (NACO). Despite reducing morbidity and mortality with the introduction of HAART, many patients fail to achieve a sustained virologic response to therapy, leading to treatment failure.

Treatment failure is an inadequate response to treatment or a lack of a sustained response based on clinical, immunological, or virological criteria [2]. Monitoring viral loads has evolved into the gold standard of care for determining ART efficacy [3]. Failure or delayed identification of a failing regimen may lead to drug resistance, toxicity, increased morbidity, and mortality [4,5]. However, it has been difficult for regions with inadequate resources to diagnose treatment failure correctly and promptly.

The present study was undertaken to study the clinical and immunological profile of first-line ART failure patients and to explore any relationship of treatment failure with the type of ART, duration of first-line ART, and adherence among HIV-infected patients with treatment failure (immunological and clinical) to first-line ART attending tertiary care hospital of north-east India.

## Materials and methods

The current hospital-based observational study was conducted among HIV-infected patients with treatment failure (immunological and clinical) to first-line ART attending ART plus center and outdoor patient department (OPD) and indoor patient department (IPD) of the Department of Medicine at the Gauhati Medical College and Hospital (GMCH) during the period July 1, 2019, to June 30, 2020.

Inclusion and exclusion criteria

HIV/AIDS patients aged above 15 years with first-line ART treatment failure (immunological and clinical) willing to participate in the study after six months of treatment were included. HIV-infected patients under 15 years, those with first-line ART duration of less than six months, ART-naive patients, and patients on or failing second-line ART were excluded from the study. HIV-2 patients were also not included. Patients unwilling to participate in the study were also excluded.

Sample size

The sample size was determined based on the following formula:

$n\ =\ \frac{z^{2}p(1\ -\ p)}{w^{2}}$

where n is the minimum sample size, w is the estimated error (0.05), p is the estimated prevalence (6%), and z = 1.96 by assuming a 95% confidence interval, and the sample size estimated is 90.

The type of first-line ART failure was defined as a clinical, immunological, or virological failure as decided by the State AIDS Clinical Expert Panel (SACEP) meeting based on the Guidelines of Treatment Failure of NACO (NACO-2018) as follows.

Clinical Failure

A new or recurrent WHO stage 4 condition after at least six months of ART is considered a clinical failure.

Immunological Failure

A fall of CD4 count to pre-therapy baseline (or below), a 50% fall from on-treatment peak value if known, or persistent CD4 levels below 100 cells/microliter after six to 12 months of treatment is considered an *immunological *failure.

Virological Failure

Plasma viral load > 1000 copies/ml is considered a virological failure.

Adherence calculation

Adherence was calculated as per the formula mentioned below:

$Adherence\ =\frac{\ Total\ number\ of\ pills\ the\ patient\ had\ actually\ taken}{Pills\ the\ patient\ should\ have\ taken\ in\ that\ time\ period} \times\ {100}$

which is also equal to:

$Adherence = \frac{Number\ of\ pills\ given\ to\ the\ patient\ -\ Number\ of\ pills\ balanced\ in\ the\ bottle}{Number\ of\ pills\ the\ patients\ should\ have\ taken} \times {100}$

Data were collected on the patient's demographics, medical history, clinical examination, and laboratory parameters in a preformed and pretested proforma.

Statistical analysis

Data were analyzed with Windows MS Excel (Microsoft Corporation, Redmond, Washington) and Statistical Package for the Social Sciences (SPSS) version 21 (IBM Corp., Armonk, NY). The distribution of categorical data was described as count and percentages, while continuous data were presented as mean and standard deviation (SD). Association between categorical variables was tested using a chi-square test, considering a p-value < 0.05 as significant.

Ethical issues

Ethical approval was obtained from the Ethics Committee of Gauhati Medical College, Guwahati, with Ref. #: MC/190/2007/Pt-II/MAR-2019/PG/54. Before collecting the data, informed consent was taken from the patients. Complete privacy was maintained for the patient’s data as per the NACO guideline.

## Results

A total of 90 HIV-infected patients with treatment failure (immunological and clinical) to first-line ART attending ART Plus Center and OPD and IPD of medicine were included in this study.

Among the first-line ART failure patients (immunological and clinical), the maximum failure rates were seen in the 30-40 age group in 38 (42.2) patients. The mean age of failure was 37.72 years. Out of the 90 patients, 64 (71%) were males with a male-to-female ratio of 2.46:1. The median BMI was 21.94, with 70 (77.8%) out of 90 patients being of average weight (Table 1).

**Table 1:** Demographic profile of HIV/AIDS patients with first-line ART failure HIV: Human immunodeficiency virus; AIDS: Acquired immunodeficiency syndrome; ART: Antiretroviral therapy.

Characteristics	Categories	Number of patients (%)
Age group (years)	10-20	5 (5.6)
	20-30	14 (15.5)
	30-40	38 (42.2)
	40-50	25 (27.8)
	50 and above	8 (8.9)
	>60	0 (0.0)
Sex	Male	64 (71.1)
	Female	26 (28.9)
BMI	Underweight (<18.5)	7 (7.8)
	Regular (18.5-24.9)	70 (77.8)
	Overweight (25–29.9)	13 (14.4)
	Obese (≥30)	0 (0.0)

The data showed high mean serum transaminase levels among the patients. The details are shown in Table 2.

**Table 2:** Clinical profile of HIV/AIDS patients with first-line ART failure HIV: Human immunodeficiency virus; AIDS: Acquired immunodeficiency syndrome; ART: Antiretroviral therapy; ESR: Erythrocyte sedimentation rate; AST: Aspartate aminotransferase; ALT: Alanine aminotransferase.

Clinical characteristics	Mean ± SD
Hemoglobin (g/dl)	11.04 ± 1.61
Total count	6329.48 ± 2299.39
Platelet count (in lacs)	2.00 ± 0.95
ESR	47.88 ± 30.79
AST (units/liter)	35.83 ± 13.37
ALT (units/liter)	41.32 ± 16.19
Creatinine	0.72 ± 0.46

As shown in Table 3, 71 (78.9%) out of 90 patients had no opportunistic infections. Pulmonary (7/90 patients) and extrapulmonary (4/90 patients) tuberculosis (12.2%) was the most common opportunistic infection, followed by bacterial and viral pneumonia (4.4%).

**Table 3:** Distribution of opportunistic infections among ART failure patients ART: Antiretroviral therapy.

Opportunistic infections	Number of patients (%)
None	71 (78.9)
Pulmonary tuberculosis	7 (7.8)
Extrapulmonary tuberculosis	4 (4.4)
Bacterial pneumonia	2 (2.2)
Pneumocystis carinii pneumonia	2 (2.2)
Oral candidiasis	2 (2.2)
Esophageal candidiasis	1 (1.1)
Extrapulmonary cryptococcosis	1 (1.1)

Of the 90 patients, 37 (41.1%) had presented with treatment failure initially at clinical stage 3 in the present study. The median baseline CD4 was 195.58. Maximum failures were experienced by patients with baseline CD4 ranging from 100-200 cells/mm^3^ in 38 (42.2%) patients, followed by those with baseline CD4 200 cells/mm^3^ and above (Table 4).

**Table 4:** Immunological profile of the patients

Variables	Categories	Number of patients (%)
Clinical stage at baseline	Stage 1	33 (36.7)
	Stage 2	12 (13.3)
	Stage 3	37 (41.1)
	Stage 4	8 (8.9)
Baseline CD4 count (cells/mm^3^)	<100	25 (27.8)
	100-200	38 (42.2)
	≥200	27 (30.0)

Among 90 treatment failure patients, all patients had an immunological failure. Virological failure was observed in 72 (80.0%) cases (Table 5).

**Table 5:** Type of failure

Type of failure	Number of patients (%)
Immunological	90 (100.0)
Clinical	8 (8.9)
Virological	72 (80.0)

**Figure 1:**
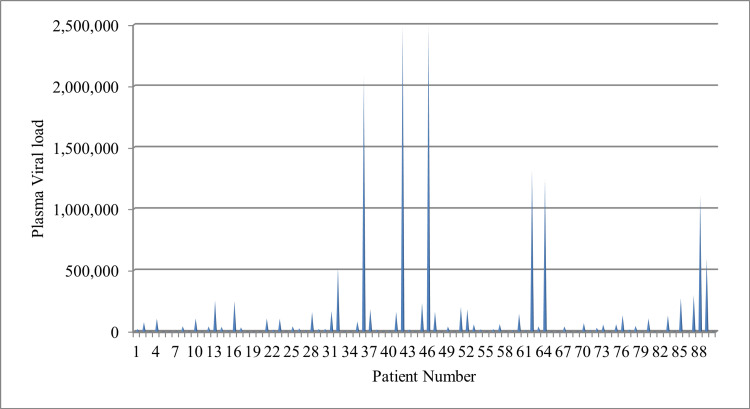
Plasma viral load of HIV/AIDS patients with first-line ART failure (clinical and immunological) HIV: Human immunodeficiency virus; AIDS: Acquired immunodeficiency syndrome; ART: Antiretroviral therapy.

The median viral load was 139646 copies/milliliter. Among the 90 ART failure patients, 44 (48.9%) had a viral load between 1,000-100,000 copies/milliliter, and 28(31.1%) had a viral load above 100,000 copies/milliliter (Figure 1).

Among the 90 first-line ART failure patients (immunological and clinical), the maximum failure rates were observed with the TLE regimen in 49 (54.5%) patients, followed by the ZLN regimen in 30 (33.3%) patients. TLN regimen had the lowest failure rate (2.2%). Maximum failures were seen in the duration of 24-30 months. The median time of failure was 37 months. Almost 19% (17/90 patients) of failures occurred after 48 months of treatment. Maximum failure was seen in the patients (38/90 patients) with adherence below 90% (Table 6).

**Table 6:** Regimen, duration, and adherence of first-line ART ART: Antiretroviral therapy; ALE: Abacavir + Lamivudine + Efavirenz; TLE: Tenofovir + Lamivudine + Efavirenz; TLN: Tenofovir + Lamivudine + Nevirapine; ZLE: Zidovudine + Lamivudine + Efavirenz; ZLN: Zidovudine + Lamivudine + Nevirapine.

Variables	Categories	No of patients (%)
ART regimen	ALE	6 (6.7)
	TLE	49 (54.5)
	TLN	2 (2.2)
	ZLE	3 (3.3)
	ZLN	30 (33.3)
Duration of first-line ART (months)	6-12	0 (0.0)
	12-18	10 (11.1)
	18-24	16 (17.8)
	24-30	38 (42.2)
	30-36	5 (5.6)
	36-42	2 (2.2)
	42-48	2 (2.2)
	>48	17 (18.9)
Adherence (%)	>95	30 (33.3)
	90-95	22 (24.4)
	<90	38 (42.2)

The factors significantly associated with time to treatment failure after ART initiation were age at ART initiation, plasma viral load, and adherence (p-value < 0.05). A significant linear trend (p-value < 0.05) was observed between age at ART initiation and time to treatment failure, indicating a delay in treatment failure (>24 months) among those aged more than 30 years. ART failure occurred significantly early (≤42 months) among those having plasma viral load >1000 copies/milliliter (p-value < 0.05). Poor treatment adherence was also considerably associated with early treatment failure (≤42 months). The details are shown in Table 7.

**Table 7:** Factors associated with time to ART failure ART: Antiretroviral therapy. * p-value for Chi-square for trend.

Factors	Categories	Time to ART failure			p-value for Chi-square
		24 months	24-42 months	>42 months	
Age group (years)	10-20	4 (12.1)	1 (2.6)	0 (0.0)	0.03*
	20-30	8 (24.2)	6 (15.8)	0 (0.0)	
	30-40	9 (27.3)	18 (47.4)	11 (57.9)	
	40-50	9 (27.3)	11 (28.9)	5 (26.3)	
	>50	3 (9.1)	2 (5.3%)	3 (15.8)	
Sex	M	25 (75.8)	28 (73.7)	11 (57.9)	0.35
	F	8 (24.2)	10 (26.3)	8 (42.1)	
Baseline CD4	<100	9 (27.3)	13 (34.2)	4 (21.1)	0.52
	100-200	13 (39.4)	13 (34.2)	11 (57.9)	
	>200	11 (33.3)	12 (31.6)	4 (21.1)	
Plasma viral load	<1000	3 (9.1)	5 (13.2)	10 (52.6)	<0.01
	1000-100000	18 (54.5)	19 (50.0)	7 (36.8)	
	>100000	12 (36.4)	14 (36.8)	2 (10.5)	
Adherence	>95	8 (24.2)	7 (18.4)	15 (78.9)	<0.01
	90-95	5 (15.2)	13 (34.2)	4 (21.1)	
	<90	20 (60.6)	18 (47.4)	0 (0.0)	

## Discussion

Managing first-line ART failure is a primary concern for HIV programs. A higher risk of morbidity and mortality is linked to continuing a failing first-line treatment regimen. Additionally, the emergence of medication resistance restricts the future development of effective and tolerable regimens. The present study was undertaken to study the clinical and immunological profiles of first-line ART failure patients and to explore any relationship between treatment failure with the type of ART, duration of first-line ART, and adherence.

The maximum number of patients with first-line ART failure (immunological and clinical) was found to be in the age group of 30-40 years (42.2%), with a mean failure age of 37.72 years. The finding suggests that older patients are less likely to fail the first-line regimen. Similar studies observed a lower incidence of treatment failure among the older age group after adherence adjustment for therapy [6]. A significant association between virologic failure and age below 40 was also documented [7,8]. However, according to a few other researches, aging is significantly linked to treatment failure, which may be because older age groups have deficient immune reconstitution mechanisms [9]. The male preponderance observed among the first-line ART failure patients in the current study with a male-to-female ratio of 2.46:1 agrees with some other literature [10].

In contrast to our observations, a recent systematic review observed that female children were at higher risk of first-line ART failure than male children [11]. However, a systematic review of observational studies published between January 1998 and November 2013 revealed no sex difference in virologic or immunologic treatment outcomes [12]. The majority (77.8%) of ART failure patients had normal BMIs with a median BMI of 21.9, which agrees with other studies [6,13,14]. The biochemical investigations showed high mean serum transaminase levels among ART failure patients. Various studies have reported hepatotoxicity as a significant concern among patients on ART [14,15]. Opportunistic infections were present in 21.1% of ART failure patients. Tuberculosis (12.2%) was the most common opportunistic infection, followed by bacterial and viral pneumonia (4.4%). Several studies have reported tuberculosis as the most common opportunistic infection among HIV/AIDS patients in India [16].

Most of the ART failure patients were presented initially at clinical stage 3. Several authors have reported a preponderance of ART failure patients with advanced baseline clinical stage [17,18]. A recent long-term follow-up study has identified advanced clinical staging as an independent predictor of treatment failure among HIV patients [19]. The median baseline CD4 of the patients was 195.58, which agrees with similar studies [20]. Maximum failures were experienced by patients with baseline CD4 ranging from 100 to 200 cells/mm^3^ (42.2%). CD4 count is the strongest predictor of subsequent disease progression and survival. An inverse relationship between the CD4 count at baseline and the risk of disease progression and treatment failure was reported by various researchers [20-22].

Among 90 treatment failure patients, all patients had an immunological failure (100%), 72 had a virological failure (80.0%), and only eight had a clinical failure (8.9%). Previous studies on treatment failure were primarily prospective, including failure and responder cases. Hence, the present study could not draw a conclusive parallel comparison of failure type.

However, in a study conducted among 54 patients with first-line treatment failure (immunological and clinical), 48 (88.9%) had immunological failure, six (11.1%) had clinical failure, and 37 (68.5%) had virological failure [23].

In our study among nucleoside/nucleotide reverse transcriptase inhibitor (NRTI) drug regimens, most (56.6%) of the first-line ART failure patients were on a tenofovir-based regimen. While among non-nucleoside reverse transcriptase inhibitor drugs (NNRTIs), first-line ART failure was mostly encountered among patients on an efavirenz-based regimen (64.5%). Previous studies have primarily compared the effectiveness between efavirenz-based HAART and protease inhibitor (PI)-based HAART, suggesting a consistent result of higher effectiveness of the efavirenz-based regimen [24]. The higher incidence of failure rates with triple NRTI regimen than with dual-class regimen (NRTI + NNRTI) is documented [2]. A recent study reported a significant association between including a PI in the first regimen and switching to a second-line treatment [22]. As the use of a PI-based regimen as first-line HAART is limited to exceptional circumstances as per NACO guidelines and infection with HIV-2 has been excluded from the present study, any case of treatment failure with a PI-based regimen could not be identified. Several studies have reported more failure in patients on stavudine and zidovudine-based NRTI regimens than tenofovir-based regimens, whereas NNRTIs mainly reported treatment failures among patients on nevirapine-based regimens in those studies [2,16,25]. In the present study, there were no patients on a stavudine-based regimen. This was because stavudine was phased out as the first-line ARV due to its toxicities, and there has been a gradual major switch to tenofovir due to its better safety profile.

Among the first-line ART failure patients (immunological and clinical), maximum failure was seen in the duration of 24-30 months, with a median time of failure of 37 months. With the early and prompt diagnosis per NACO operational guidelines, the median time of switching to the second line has been delayed as observed in the present study, which agrees with another Indian study [25]. Also, with the increasing focus on the enhanced adherence counseling (EAC) and the choice of well-designed ARV regimens, the median time of first-line ART failure has improved.

Adherence is the most significant patient-enabled predictor of treatment outcomes for the patients on HAART. WHO recommends at least 95% adherence to ART to avoid the emergence of resistant strains. In our study, among the first-line ART failure patients (immunological and clinical), maximum failure was seen in the patients with adherence of <90%. These findings were almost like another study. Previous research has identified inadequate adherence as an independent predictor of first-line ART treatment failure [26]. A recent study from India stated that high costs, alcoholism, choosing non-allopathic drugs, and depression were the most frequent causes of inadequate adherence and treatment termination. Missed doses were attributed to various factors, including feeling well, depression, forgetfulness, and a busy schedule [27]. The remarkable improvements in HIV-related health indices could eventually be negated by nonadherence. Due to high viral mutation rates and short drug half-lives in nonadherent patients, resistant virus strains emerge, significantly narrowing the range of available antiviral treatments. Therefore, even if adherence is increased, poor adherence may encourage the selection of mutants that are no longer sensitive in addition to immediate increases in plasma HIV RNA.

The global HIV response, with coordinated political and financial commitment, has made substantial progress toward its goals of reaching critical populations and other marginalized groups, removing barriers to treatment caused by poverty, integrating communities in service delivery and decision-making processes, and placing the right to health at the forefront [28]. Enhancement of pandemic preparedness and response has been a topic of discussion across the globe as a result of the recent COVID-19 pandemic and the expectation of future pandemic threats. The HIV response is an excellent representation of a worldwide health endeavor that has succeeded in significantly advancing pandemic preparedness and response [29]. Although the number of new HIV infections and AIDS-related fatalities has significantly declined since the epidemic's peak, there has not been much improvement in reducing new infections in the last decade. [28] Minimizing stigma among HIV-infected patients and those who are at risk of the disease, expanding the HIV workforce, reducing detrimental socioeconomic determinants of health, and recommitting and reinvesting in health are the priority areas that are to be considered globally for HIV elimination strategies [30].

Limitation

The results of this observational study, which was limited to one ART center, may only apply to some. To draw a firm conclusion, further prospective research with a bigger sample size in a larger community and several ART centers is required.

## Conclusions

Younger male patients with normal BMIs had a higher propensity for treatment failure. Low baseline CD4 count and poor adherence were influential in the occurrence of treatment failure. Compared to other NRTIs and NNRTIs, the incidence of first-line ART failure was higher with tenofovir- and efavirenz-based regimens. The median time of first-line ART failure has been postponed due to the development of newer, more potent ARV medications.
